# Evaluation of Simultaneous Growth of *Escherichia coli* O157:H7, *Salmonella* spp., and *Listeria monocytogenes* in Ground Beef Samples in Different Growth Media

**DOI:** 10.3390/foods13132095

**Published:** 2024-07-01

**Authors:** José Mário Sousa, Ana Barbosa, Daniela Araújo, Joana Castro, Nuno Filipe Azevedo, Laura Cerqueira, Carina Almeida

**Affiliations:** 1 CEB—Centre of Biological Engineering, LIBRO—Laboratory of Research in Biofilms Rosário Oliveira, Campus de Gualtar, University of Minho, 4710-057 Braga, Portugal; mariosousa20@outlook.com (J.M.S.); daniela.araujo@iniav.pt (D.A.); joana.castro@iniav.pt (J.C.); carina.almeida@iniav.pt (C.A.); 2 LEPABE—Laboratory for Process Engineering, Environment, Biotechnology and Energy, Faculty of Engineering, University of Porto, Rua Dr. Roberto Frias, 4200-465 Porto, Portugal; up202204092@edu.fe.up.pt (A.B.); nazevedo@fe.up.pt (N.F.A.); 3 ALiCE—Associate Laboratory in Chemical Engineering, Faculty of Engineering, University of Porto, Rua Dr. Roberto Frias, 4200-465 Porto, Portugal; 4 INIAV, IP—National Institute for Agrarian and Veterinary Research, Rua dos Lagidos, Lugar da Madalena, Vairão, 4485-655 Vila do Conde, Portugal; 5 LABBELS—Associate Laboratory, 4710-057 Braga, Portugal

**Keywords:** foodborne pathogens, growth media, culture media comparison, pathogen detection

## Abstract

Several multiplex approaches for the simultaneous detection of pathogens in food have been developed in recent years, but the use of a single enrichment medium remains a problem. In this study, six enrichment broths (five non-selective media, tryptic soy broth (TSB), brain heart infusion broth (BHI), buffered peptone water (BPW), universal pre-enrichment broth (UPB), no. 17 broth, and a selective, *Salmonella Escherichia Listeria* broth (SEL)), were studied for the simultaneous detection of *E. coli* O157:H7, *Salmonella* spp., and *L. monocytogenes*, to validate the suitable enrichment broth to be used for the detection methods. Different ratios of *E. coli* O157:H7, *Salmonella* spp., and *L. monocytogenes* were used. Almost all non-selective broths evaluated in this study showed similar growth parameters and profiles among each other. The only selective enrichment broth under analysis (SEL) showed distinct growth features compared to the non-selective media, allowing for a slower but balanced growth of the three pathogens, which could be beneficial in preventing the overgrowth of fast-growing bacteria. In addition, when tested in ground beef samples, SEL broth seems to be the most distinctive medium with a balanced growth pattern observed for the three pathogens. Overall, this study is intended to provide the basis for the selection of suitable enrichment broths according to the technology detection to be used, the desired time of enrichment, and the expected balanced concentration of pathogens.

## 1. Introduction

Foodborne diseases represent an important cause of morbidity and mortality that have a negative impact on public health, the economy, and society worldwide [1]. Foodborne pathogens are responsible, in the US, for nearly 48 million cases of foodborne illness each year, which result in 128,000 hospitalizations and 3000 deaths [2,3,4]. Pathogens such as *Salmonella* spp., *Listeria monocytogenes*, Shiga-toxin-producing *Escherichia coli* (STEC) O157:H7, *Campylobacter* spp., *Yersinia* spp., and *Shigella* spp. are considered to be the major bacterial foodborne pathogens [4,5]. From these, *E. coli* O157:H7, *Salmonella* spp., and *L. monocytogenes* are probably the most studied and surveyed due to their high infectious potential; the severity of the illness caused; the relatively high prevalence of the population; and their capacity to contaminate a wide range of food samples [6]. Given the complexity of global food supply chains, the application of systems analysis methods to food security is of paramount importance. The standardization of the food analysis protocols can increase the simplicity of analysis, which would drive the implementation of food security measures in the food supply chain.

Technology is evolving towards the more rapid and simple detection of foodborne pathogens, allying simplicity, accuracy, and time-to-result to surpass the socio-economic problems associated with food contamination. Immunoassays, polymerase chain reaction (PCR), biosensors, and fluorescence in situ hybridization (FISH)-based techniques are some examples of new approaches to pathogen detection [7,8,9,10]. However, all the food sampling protocols need to be well defined to optimize the detection method performance.

Traditionally, the detection methods require the use of an enrichment step to increase the target pathogen concentration to detectable levels in a short period of time, usually not exceeding 24 h [11,12], to avoid the procedure being extended to an additional working day. For PCR-based applications and immunological and biosensor assays, the target pathogen concentration should reach a detection limit of around 10^3^–10^4^ colony-forming units per millilitre (CFUs/mL) [9], and for the FISH technology, the limit is about 10^3^–10^6^ CFUs/mL [13,14]. The enrichment protocols are also important to recover injured or stressed cells that result from food processing and storage [15] and to decrease the interference of inhibitors [8,16,17], improving the efficiency of the target pathogen detection [18,19].

Allied with this, the simultaneous detection of foodborne agents is advantageous because it reduces the associated costs and labour, allowing for the testing of the same food sample for contamination with different pathogens at the same time [8,15]. The most often used media for the simultaneous enrichment of foodborne pathogens (especially *E. coli* O157:H7, *Salmonella* spp., and *L. monocytogenes*) include tryptic soy broth (TSB) [20,21], brain heart infusion broth [22], buffered peptone water (BPW) [23], universal pre-enrichment broth (UPB) [24], no. 17 broth [25], and the selective *Salmonella Escherichia Listeria* broth (SEL) [16]. However, their performances have never been compared extensively and some media compositions may not be able to provide a balanced growth of different pathogens, which might hide some of the populations in the analysis [26]. This is especially important for culture-based detection methods, where the dominant population forms a lawn that masks the results [16].

In this study, the six enrichment broths mentioned were studied for the simultaneous detection of *E. coli* O157:H7, *Salmonella* spp., and *L. monocytogenes*, in order to validate the suitable enrichment broths to be used. The media were then evaluated in terms of growth profile and kinetic parameters in single and co-cultures. Further assays with raw ground beef samples were carried out to evaluate the performance of enrichment media in the presence of a natural competing microbiota.

## 2. Materials and Methods

### 2.1. Bacterial Strains and Culture Conditions

To study the kinetic parameters in different enrichment media, three different food-borne bacterial species were included and distributed into group I and group II. Each group contains one strain of each pathogen in analysis. The strains belong to the Spanish Type Culture Collection, Spain (CECT); South Georgia State College Collection, Georgia, USA (SGSC); or the National Collection of Type Cultures, UK (NCTC). Group I comprised *Escherichia coli* O157:H7 CECT 4783, *Salmonella enterica* serovar Enteritidis SGSC 2476, and *Listeria monocytogenes* CECT 4031T; group II included *E. coli* O157:H7 CECT 4267, *Salmonella* serovar Typhimurium NCTC 12416, and *L. monocytogenes* 747. These two groups were used for the single-culture assays. For co-culture experiments, only group II of strains was used. All strains were maintained on tryptic soy agar (Liofilchem, Roseto degli Abruzzi, Italy) at 37 °C.

### 2.2. Enrichment Media

Six media were selected, namely, tryptic soy broth (TSB) (Liofilchem), buffered peptone water (BPW) (Liofilchem), brain heart infusion broth (BHI) (Liofilchem), universal pre-enrichment broth (UPB) (Dehydrated Culture Media: Universal Preenrichment Broth) (Becton Dickinson, Franklin Lakes, NJ, USA), no. 17 broth, and *Salmonella Escherichia Listeria* broth (SEL) (Table 1). All media were already purchased in a ready-to-use form, except SEL and no. 17 broth. SEL was prepared with a base medium (buffered listeria enrichment broth, BLEB) (Liofilchem) to which the selective agents were added, 0.01 g/L Acriflavine (Sigma-Aldrich, St. Louis, MO, USA), 0.05 g/L Cycloheximide (Fluka, Buchs Switzerland), 0.002 g/L Nalidixic acid (Applichem, Darmstadt, Germany), and 0.05 g/L Fosfomycin (Sigma-Aldrich, St. Louis, MO, USA), as described in Kim and Bhunia [26]. No. 17 broth was prepared according to the formulation described by Kawasaki and colleagues [25] with some modifications suggested by Garrido and colleagues [18] and by Omiccioli and coworkers [27], and it included 10 g/L tryptose (Liofilchem), 5 g/L beef extract (Merck, Darmstadt, Germany), 5g/L yeast extract (Liofilchem), 5 g/L sodium chloride (Sigma-Aldrich), 19.3 g/L disodium phosphate (Sigma-Aldrich), and 3.4 g/L monopotassium phosphate (Sigma-Aldrich).

### 2.3. Evaluation of Growth Kinetics in Single Cultures

For the determination of growth kinetic parameters (exponential growth rate and doubling time) in a single culture, a protocol similar to that described by Garrido and colleagues [18] was followed. A loopful of each bacterium was inoculated into 20 mL of TSB and the cultures were allowed to grow at 37 °C, for approximately 18 h, with an agitation of 120 rpm (Stuart Scientific, Stone, UK). An amount of 25 µL of the stationary phase culture was then inoculated in 20 mL of each enrichment medium under analysis and incubated until the stationary growth phase, at 37 °C, in a shaking incubator at 120 rpm. In the case of no. 17 broth, the temperature used was 35 °C as recommended by Kawasaki and colleagues [25]. The growth was monitored through measurements of optical density at 600 nm (OD_600nm_) overtime, with a microplate reader device (Tecan, Männedorf, Switzerland). All the experiments were repeated at least twice.

### 2.4. Evaluation of Growth Kinetics in Co-Cultures

Growth kinetic parameters in co-cultures were determined as described before [26], with minor modifications. The experiments were performed using group II of strains described in Section 2.1. Each experiment involved the co-inoculation of the pathogens in different proportions. In addition, herein, only four growth media were tested here: TSB, UPB, no. 17, and SEL.

For this evaluation, different concentration ratios of the three pathogens were used as inoculum. In experiment I, equal concentrations of the three pathogens (*E. coli* O157:H7, *Salmonella* spp., and *L. monocytogenes*) were inoculated in a 1:1:1 proportion, with ~1 CFU/mL of each pathogen. In the other experiments, *E. coli*, *Salmonella* spp., and *L. monocytogenes* ratios were as follows: experiment II—1:1000:10; experiment III—10:1:1000; and experiment IV—1000:10:1.

Briefly, pre-inocula of *E. coli*, *Salmonella* spp., and *L. monocytogenes* were prepared by inoculating a loopful of each bacterium into 20 mL of TSB. Cultures were allowed to grow at 37 °C, overnight (~18 h), with an agitation of 120 rpm (Stuart Scientific). Afterward, the OD_600nm_ of each pre-inoculum was measured and adjusted to attain a concentration of 10^8^ colony-forming units per millilitre (CFUs/mL). Serial ten-fold dilutions were made in phosphate-buffered saline (PBS, 8 g/L NaCl, 0.2 g/L KCl, 1.44 g/L Na_2_HPO_4_, 0.2 g/L KH_2_PO_4_, pH 7.2) and then used to inoculate 20 mL of the enrichment media under analysis, in the proportions described above (experiment I, II, III, and IV). Inoculum concentrations were confirmed by plating the appropriate dilutions on tryptic soy agar (TSA) plates. Next, each enrichment medium was incubated at 37 °C (35 °C for no. 17 broth), for 24 h, in a shaking incubator at 120 rpm. Samples were taken at different incubation times for 24 h, and cell counts for each pathogen were determined by plating the cells (and the adequate ten-fold dilution in PBS), in triplicate, onto plates with the appropriate selective agar. MacConkey agar (Liofilchem) was used for *E. coli* O157:H7 and *Salmonella* spp., and Oxford Listeria agar (Liofilchem) was used for *L. monocytogenes*. Lastly, the morphology of bacteria was accessed by the Stereo-Microscope SZ61TR (Olympus, Hamburg, Germany). These experiments were repeated twice for each group of strains.

### 2.5. Kinetic Parameters Determination

Calculations for the determination of specific growth rates and doubling times were determined through the equation of exponential growth using GraphPad Prism version 8^®^ software (GraphPad Software, San Diego, CA, USA) both in pure and co-culture assays. The equations for co-culture assays are presented in Table S1. The exponential specific growth rate and doubling time were determined through the linear regression determination. For pure culture assays, growth parameters were calculated through OD_600nm_ versus time graphical data; while for co-culture assays, graphics of log_10_ CFUs/mL versus time were used.

### 2.6. Performance on Ground Beef Samples

In order to evaluate real conditions of enrichment, 25 g of ground beef samples acquired on the previous day from a local retailer (Pingo Doce, Braga) were artificially contaminated with low levels of the three pathogens of group II described in Section 2.1, as previously described [31], in a proportion of 1:1:1, with 1 CFU/mL. Of note, a non-inoculated batch was also kept and analysed simultaneously as an experimental control.

For the artificial contamination of ground beef samples, pre-inocula of *E. coli* O157:H7 CECT 4267, *Salmonella* serovar Typhimurium NCTC 12416, and *L. monocytogenes* 747 (group II of strains) were prepared as described above. The inoculums were then adjusted to the desired concentration, confirmed by plating the bacterial suspensions on TSA. Twenty-five grams of artificially contaminated ground beef was mixed in aseptic conditions with 225 mL of pre-warmed enrichment medium, in 1000 mL flasks, and incubated at 37 °C for 24 h in a shaking incubator at 120 rpm. Assays involving no. 17 broth were performed at 35 °C. Samples were taken at different incubation times for 24 h. Cell counts for each pathogen were determined by plating the cells in appropriate selective agar: CT-SMAC agar (Thermo Scientific, Waltham, MA, USA) for *E. coli* O157:H7 (Thermo Scientific, Waltham, MA, USA) for *Salmonella* spp. and Oxford Listeria agar for *L. monocytogenes*. The total viable cell counts were assessed using TSA media through the following process: samples were first serially diluted in sterile saline to obtain a range of dilutions. Aliquots of each dilution were then spread onto TSA plates. The plates were incubated at 37 °C for 24 to 48 h to allow for colony formation. After incubation, the colonies were counted manually. The number of colonies was then multiplied by the dilution factor to calculate the total viable cell counts, expressed as colony-forming units per millilitre (CFUs/mL). This method ensures the accurate and reproducible quantification of viable cells in the sample.

### 2.7. Statistical Analysis

The data were analysed using the statistical package GraphPad Prism version 8 by one-way ANOVA, with multiple comparisons by Tukey’s multiple comparisons test or Bartlett’s test. For non-linear curves, a non-parametric analysis was performed by using the Kruskal–Wallis test. Values with *p* < 0.05 were considered statistically significant.

## 3. Results and Discussion

### 3.1. Evaluation of Growth Kinetics in Single Cultures

**Figure 1:**
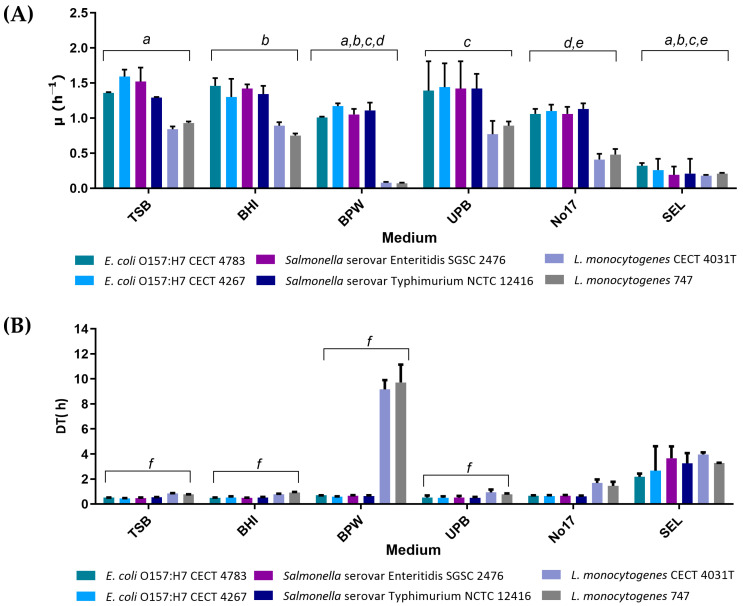
Growth kinetics in single cultures. (**A**) Values of µ—Exponential Growth Rate (unit: h^−1^). (**B**) DT—Doubling Time (unit: h) on single culture assays for the six enrichment media under analysis. ^a^ Indicates that there is a statistical difference in values of µ (*p* < 0.05) between TSB and BPW; TSB and SEL. ^b^ Indicates that there is a statistical difference in values of µ (*p* < 0.05) between BHI and BPW; BHI and SEL. ^c^ Indicates that there is a statistical difference in values of µ (*p* < 0.05) between UPB and BPW; UPB and SEL. ^d^ Indicates that there is a statistical difference in values of µ (*p* < 0.05) between BPW and no. 17. ^e^ Indicates that there is a statistical difference in values of µ (*p* < 0.05) between no. 17 and SEL. ^f^ Indicates that there is a statistical difference in values of DT (*p* < 0.05) between BPW and TSB; BPW and BHI; and BPW and UPB. Error bars represent standard deviation. Abbreviations: tryptic soy broth (TSB); brain heart infusion broth (BHI); buffered peptone water (BPW); universal pre-enrichment broth (UPB); no. 17 broth; and *Salmonella Escherichia Listeria* broth (SEL).

The growth profile of *E. coli* O157:H7, *Salmonella* spp., and *L. monocytogenes* was determined individually in each of the six selected enrichment broths through OD_600nm_ measurements over time as shown in Figure 1. The assays in single cultures intended to identify the media with the best features regarding the exponential growth rate (µ) and doubling time (DT).

As it is possible to observe in Figure 1A, TSB, BHI, and UPB generally presented the highest values of µ and the lowest values of DT in all strains analysed. The results for these three media are very similar (*p* > 0.05), which might be explained by their highly nutritive nature [27]. In addition, the results are very similar between the two strains of each species, and no statistical difference was found (*p* > 0.05). Among the tested conditions, overall, the highest µ values were observed in *E. coli* O157:H7 CECT 4783, *E. coli* O157:H7 CECT 4267, *Salmonella* serovar Enteritidis SGSC 2476, and *Salmonella* serovar Typhimurium NCTC 12416, while the lowest µ value was observed for *L. monocytogenes* CECT 4031T and *L. monocytogenes* 747. It is also important to highlight that in the case of UPB, the high content of salts (buffering ability) helps the recovery of sub-lethally injured cells [30]. Although the study here was carried out with single cells in the exponential phase, this may be advantageous in food matrices where pathogens are often present in low numbers and under sub-lethal conditions.

The results obtained with BPW and no. 17 broth show that these media perform well for *E. coli* and *Salmonella* spp. but slightly worse than TSB, BHI, or UPB. Concerning the *L. monocytogenes* values in BPW, the results are considerably different. The µ values of *L. monocytogenes* are extremely low and, consequently, the DT values are very high (Figure 1B). These results are probably related to the fastidious behaviour of *L. monocytogenes* [32] and the lower content of nutrients of BPW compared to the other rich broths. It is also possible to observe that even in highly nutritive broths (TSB, BHI, UPB, and no. 17), *L. monocytogenes* always show lower µ and higher DT due to the fastidious behaviour referred to above.

SEL is the only selective enrichment broth in the analysis. This medium was developed [26] as a new selective enrichment broth for simultaneous growth of *E. coli* O157:H7, *Salmonella* spp., and *L. monocytogenes*, with promising results for enriching the mixed cultures of these three pathogens. The results show that µ values obtained with SEL assays are much lower compared to other broths (Figure 1A). This feature is probably related to the selective nature of the medium [33]. The presence of antibiotics and limitative agents appears to interfere with the growth, making it slower and less pronounced. An interesting trait is that SEL appears to balance the growth of the three pathogens once they reveal identical values of µ and DT.

### 3.2. Evaluation of Growth Kinetics in Co-Cultures

After selecting the media best fitted to single pathogen cultures, their performance in co-cultures was evaluated. Since no statistical differences were found between the bacterial strains belonging to the same bacterial species, as mentioned above, only group II strains were selected here.

For further evaluation of the performance of enrichment broths in co-cultures, four media were selected: TSB, UPB, no. 17, and SEL. BHI was excluded due to its high similarities with TSB and UPB in terms of kinetic parameters, while BPW was discarded due to the poor results obtained for *L. monocytogenes*.

TSB, UPB, no. 17, and SEL were inoculated at different ratios to better evaluate the influence of a dominant species on the performance of the others. In experiment I, equal concentrations of the three pathogens (*E. coli*, *Salmonella* spp., and *L. monocytogenes*) were inoculated in a 1:1:1 proportion (with ~1CFU/mL of each pathogen). In the other experiments, *E. coli*, *Salmonella* spp., and *L. monocytogenes* ratios were as follows: experiment II—1:1000:10; experiment III—10:1:1000; and experiment IV—1000:10:1.

**Figure 2:**
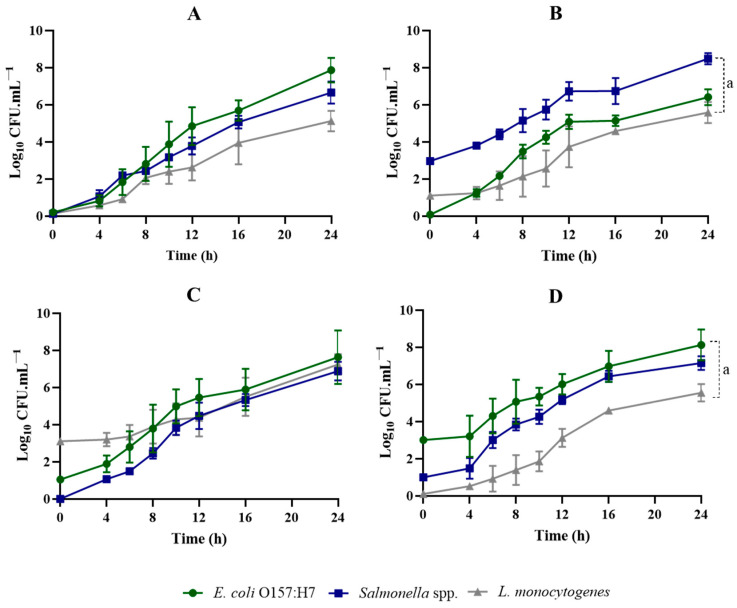
Growth curves for *E. coli* O157:H7, *Salmonella* spp., and *L. monocytogenes* co-cultured in SEL, at different pathogen ratios. *E. coli* O157:H7/*Salmonella* spp./*L. monocytogenes*. (**A**) Ratio of 1:1:1. (**B**) Ratio of 1:1000:10. (**C**) Ratio of 10:1:1000. (**D**) Ratio of 1000:10:1. ^a^ Indicates that there is a statistical difference in values of µ (*p* < 0.05) between *E. coli* O157:H7 and *L. monocytogenes*.

**Figure 3:**
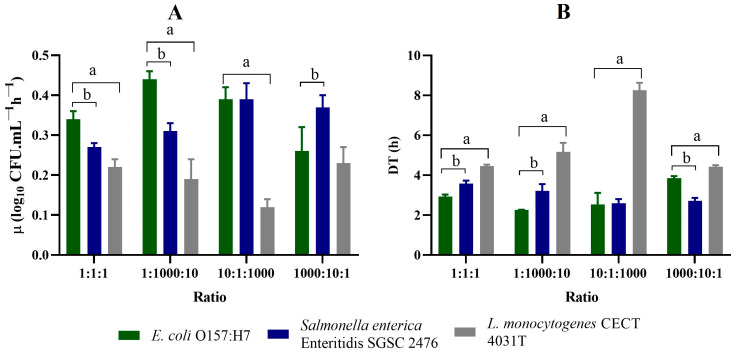
Growth kinetics in co-cultures. (**A**) Values of µ—Exponential Growth Rate (unit: log_10_ CFUs mL^−1^h^−1^). (**B**) DT—Doubling Time (unit: h) (**B**) for *E. coli* O157:H7, *Salmonella* spp., and *L. monocytogenes* co-cultured in SEL, at different pathogen ratios. *E. coli* O157:H7/*Salmonella* spp./*L. monocytogenes* ratio of 1:1:1, 1:1000:10, 10:1:1000, and 1000:10:1. ^a^ Indicates that there is a statistical difference in values of µ (*p* < 0.05) between *E. coli* O157:H7 and *L. monocytogenes*. ^b^ Indicates that there is a statistical difference in values of µ (*p* < 0.05) between *E. coli* O157:H7 and *Salmonella* spp.

Regarding the growth patterns of the three pathogens in SEL broth (Figure 2 and Figure 3), there were considerable differences when compared with those of TSB, UPB, and no. 17 (Figure S1, Figure S2, and Figure S3, respectively).

The selective enrichment broth under examination (SEL) exhibited unique growth characteristics (Figure 2) compared to the non-selective media (Table S2).

It facilitated slower yet well-balanced growth of all three pathogens, which could prove beneficial in preventing the dominance of fast-growing bacteria.

Looking at the growth patterns for all the proportions accessed (Figure 2), it is possible to observe that SEL does not have the same ability to recover low numbers of cells, compared to TSB, UPB, or no. 17 broth (Table S2). As expected, SEL has demonstrated to be much slower in recovering both lower and higher levels of the three pathogens. At the end of the 24 h assay, the *E. coli* and *Salmonella* spp. concentrations hardly reached values around 10^8^ CFUs/mL, even for higher inoculum concentrations. In the case of *L. monocytogenes*, at the end of the 24 h assay, it reached values around 10^5^ CFUs/mL and up to 10^6^ when inoculated with higher concentrations. The presence of selective agents appears to retard and diminish the growth of all the target bacteria (Figure 3 and Table S2). Nonetheless, selective agents can constitute simultaneously the main limitation factor and the main advantage of SEL, when using food samples. This effect will be further evaluated in the next step.

**Figure 4:**
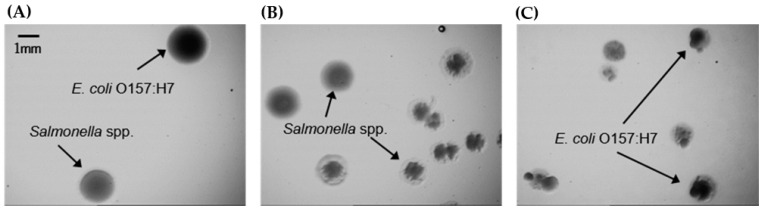
Morphology of some *E. coli* O157:H7 and *Salmonella* spp. colonies in McConkey agar plates at the early hours of SEL assays. (**A**) Regular morphology of *E. coli* O157:H7 and *Salmonella* spp. (**B**) Regular and irregular morphology of *Salmonella* spp. (**C**) Irregular morphology of *E. coli* O157:H7. The images were acquired by using the Stereo-Microscope SZ61TR.

Another interesting feature of the growth patterns in the SEL medium is the presence of some kind of adapting period (until ~4 h), where cell numbers remain at the initial inoculation level or even lower (Figure 2). In the CFU counts, it was possible to observe that some colonies of *E. coli* and *Salmonella* spp. showed irregular morphology within this period (Figure 4).

This was only observed for cells grown in SEL. With further streaking of colonies into fresh plates, the irregular colonies showed regular shape. This behaviour might be related to the induction of some level of stress in bacteria, affecting its morphology and growth in the early stages.

The growth patterns of the pathogens in TSB, at similar initial inoculation levels (Table S2), showed a similar growth behaviour to *E. coli* and *Salmonella* spp., while *L. monocytogenes* growth was slightly slower. *E. coli* and *Salmonella* spp. µ values were 0.65 ± 0.02 and 0.63 ± 0.12 log10 CFUs mL^−1^ h^−1^, respectively, while *L. monocytogenes* reached an µ value of 0.35 ± 0.03 log10 CFUs mL^−1^ h^−1^ (Table S2). These data express that *E. coli* and *Salmonella* spp. were able to grow better in TSB when compared to *L. monocytogenes*, even with similar initial concentrations. *L. monocytogenes* might be affected by some level of nutrient depletion once *E. coli* and *Salmonella* spp. are fast-growing bacteria. But, in fact, the µ and DT values observed in co-cultures are very similar to those observed in pure cultures. This means that these three species do not seem to greatly affect the growth of each other.

In experiment II (Figure S1B) the initial pathogen ratio was 1:1000:10 for *E. coli*, *Salmonella* spp., and *L. monocytogenes*, respectively. The growth profiles of *Salmonella* spp. and *E. coli* seem to be proportional to the initial inoculums, reaching a differential final concentration (at 24 h) of about 2 log_10_ CFUs/mL (Figure S1B), with *Salmonella* spp. reaching higher cell concentrations than *E. coli*, as would be expected. The growth pattern of *L. monocytogenes* is very similar to the one observed in experiment I (Figure S1A).

The observed µ and DT values do not differ significantly from the ones at experiment I (Table S2), regardless of the initial concentration of *L. monocytogenes*, which was 10 times higher than *E. coli*. *L. monocytogenes* was rapidly surpassed by the other species in terms of cell number. This behaviour has already been reported by Mellefont and co-workers [34]. However, it is important to observe that *L. monocytogenes* shows interesting behaviour when both *Salmonella* spp. and *E. coli* reach the stationary phase. It managed to maintain the cellular growth, which may indicate that the conditions that are limiting the growth of *Salmonella* spp. and *E. coli* (such as the nutrient depletion, accumulation of toxic metabolites, and higher number of cells) do not limit the growth of *L. monocytogenes*, at least until this bacterium reaches 10^6^–10^7^ CFUs/mL.

Regarding experiments III and IV, in TSB media (Figure S1C,D), the exponential growth phase parameters do not seem to be greatly affected by the initial inoculums (Table S2). Actually, when inoculated at a high concentration, *L. monocytogenes* reaches its maximum concentration (and, consequently, the stationary phase) early in the assay; but still the maximum concentration is fixed at 10^7^ CFUs/mL. It always reached lower concentrations no matter the initial concentration used.

The other non-selective media in analysis (UPB and no. 17 broth) present high similarities with the results observed for TSB (Figures S1–S3), both in terms of the growth profile and kinetic parameters (Table S2).

### 3.3. Performance on Ground Beef Samples

**Figure 5:**
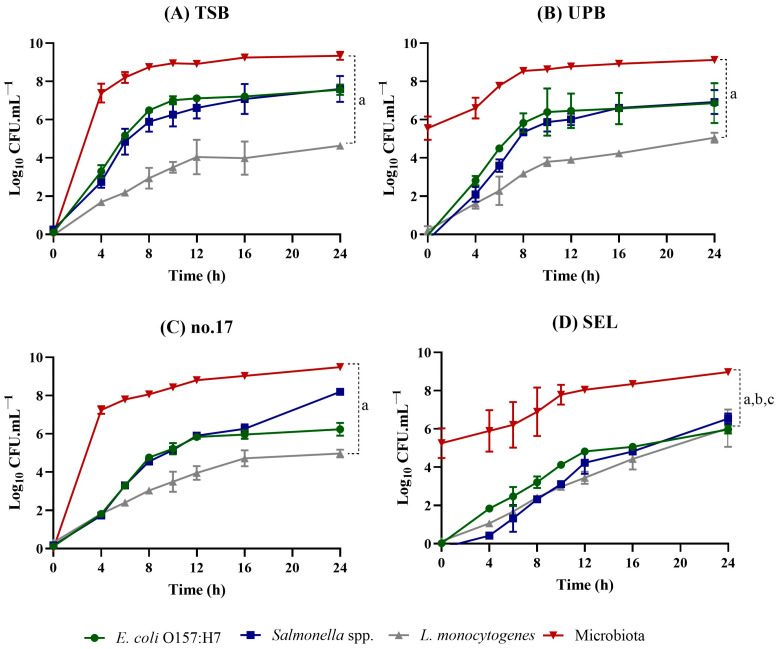
Growth curves for *E. coli* O157:H7 CECT 4267, *Salmonella* serovar Typhimurium NCTC 12416, and *L. monocytogenes* 747 in an artificially contaminated ground beef sample. (**A**) Enrichment on TSB. (**B**) Enrichment on UPB. (**C**) Enrichment on no. 17 broth. (**D**) Enrichment on SEL. ^a^ Indicates that there is a statistical difference in values of µ (*p* < 0.05) between *L. monocytogenes* 747 and microbiota. ^b^ Indicates that there is a statistical difference in values of µ (*p* < 0.05) between *Salmonella* spp. and microbiota. ^c^ Indicates that there is a statistical difference in values of µ (*p* < 0.05) between *E. coli* O157:H7 and microbiota.

In order to simulate the real conditions of pre-enrichment, with the presence of a competing microbiota, a food matrix was included. Ground beef was chosen as it is colonized by a variety of microorganisms [35,36] and it is often associated with foodborne infections caused by the three pathogens under study. Samples of 25 g of ground beef were inoculated with 1 CFU/mL of each pathogen, homogenized with 225 mL of enrichment broth, and incubated for 24 h (35 or 37 °C). CFU counts were performed using several selective media and also TSA to assess the total culturable microbiota. Results are shown in Figure 5.

It is clear that TSB, UPB, and no. 17 broth provide similar growth conditions (Figure 5A–C). The growth patterns on no. 17 broth show that the growth is slightly slower than that observed in TSB and UPB assays, probably due to the use of a lower incubation temperature (35 °C). At the end of the 24 h of enrichment, the concentration of *E. coli* and *Salmonella* spp. ranged between 10^6^ CFUs/mL and 10^8^ CFUs/mL and *L. monocytogenes* ranged from 10^4^ CFUs/mL to 10^5^ CFUs/mL in these three media. For the screening approach to be performed subsequently, the concentrations obtained following the enrichment step should provide pathogen concentrations to detectable levels. The results obtained for the three pathogens enable the application of PCR techniques or immunology assays [9]; however, to apply FISH technology, the levels of *L. monocytogenes* achieved may not be sufficient to obtain a favourable result [13]. The slow and less pronounced growth profile of *L. monocytogenes* in the presence of natural microbiota may be due to both the growth-limiting effect of a larger competing microbiota (when compared to the assay in co-cultures) and the saturation effect of the medium [37]. This behaviour of *L. monocytogenes* has been reported previously [28] for the enrichment of ground pork samples. During the enrichment, the vacant media transforms into a saturated culture, usually corresponding to a cell count of ~10^9^ CFUs/mL. When the culture reaches this value, the physical space available becomes limiting.

Regarding the enrichment in SEL broth, once again, the most distinctive feature is the balanced growth pattern observed for the three pathogens (Figure 4). The selective properties of SEL seem to only affect the overall microorganism concentration. Even though the concentration of the pathogens was balanced (between 10^5^ and 10^6^ CFUs/mL), a slower and less pronounced growth was observed. This could be a disadvantage for those detection methods with high detection levels. This might be the case with immunological techniques such as some ELISA assays, known for their low limit of detection [9,38]; several types of biosensors [38]; and some fluorescence in situ hybridization (FISH) procedures, where the detection limit is usually around 10^5^ CFUs/mL [13]. In those particular cases, the use of a rich medium might be advisable, instead of a selective one. Nonetheless, the choice of the preferred medium will depend on the properties of the detection technique to be applied.

## 4. Conclusions

Multiplex detection possesses a number of advantages, but the need for a universal enrichment step is a limitative factor. The choice of an enrichment broth depends on several factors such as the detection limit of the technique to be used, the growth capacity of the target microorganism, and, ultimately, the characteristics of the medium itself.

Regarding this, the selective medium SEL provides a slower and less pronounced growth for all three pathogens tested, which are clearly affected by the medium’s composition. This medium in fact has been demonstrated to be much slower in recovering low levels of *E. coli* O157 and *Salmonella* spp., and it also failed to recover *L. monocytogenes* up to 10^6^ CFUs/mL; however, it seems to present an advantage in preventing the overgrowth of fast-growing bacteria.

In conclusion, the present study shows that the choice of an enrichment broth is clearly a limiting factor for obtaining detection and balanced levels of the pathogens, which has implications for the detection technology to be applied in the future. Furthermore, it is important to understand the behaviour of the selective medium in other food matrixes.

## Figures and Tables

**Table 1:** Constituents of enrichment broths in analysis. Adapted from Gehring and coworkers [28].

Medium	Nutrient (s)	Buffer (s)	Salt (s)	Supplement (s)	pH	g/L H_2_O	Reference
BPW	Peptone	Phosphate	NaCl	-	7.0	20	[29]
TSB	Casein digest, soybean digest, dextrose	Phosphate	NaCl	-	7.3	30	[20]
UPB	Casein digest, soybean digest, dextrose, protease peptone, sodium pyruvate	Phosphate	NaCl, Mg_2_SO_4_, Ferric ammonium citrate	-	6.3	38	[30]
SEL	Pancreatic digest of casein, yeast extract, dextrose, soytone, sodium pyruvate	Phosphate	NaCl	Acriflavine, Cycloheximide, Fosfomycin, Nalidixic Acid	7.0	48	[26]
BHI	Brain heart infusion, casein digest, dextrose	Phosphate	NaCl	-	7.4	37	[27]
no. 17	Tryptose, beef extract, yeast extract	Phosphate	NaCl	-	7.2	48	[18]

## Data Availability

The original contributions presented in the study are included in the article, further inquiries can be directed to the corresponding author.

## Supplementary Materials

The following supporting information can be downloaded at https://www.mdpi.com/article/10.3390/foods13132095/s1, Figure S1: Growth curves for *E. coli* O157:H7, *Salmonella* spp., and *L. monocytogenes* co-cultured in TSB, at different pathogen ratios. Figure S2: Growth curves for *E. coli* O157:H7, *Salmonella* spp., and *L. monocytogenes* co-cultured in UPB, at different pathogen ratios. Figure S3: Growth curves for *E. coli* O157:H7, *Salmonella* spp., and *L. monocytogenes* co-cultured in no. 17 broth, at different pathogen ratios. Table S1: Equations for measuring the exponential growth and doubling time for TSB, UPB, no. 17 broth, and SEL for the co-culture assays. Table S2: Values of DT and µ for TSB, UPB, no. 17 broth, and SEL for the co-culture assays.
