# Supplementary material for: Evaluation of Simultaneous Growth of Escherichia coli O157:H7, Salmonella spp., and Listeria monocytogenes in Ground Beef Samples in Different Growth Media
Source: Foods. 2024 Jul 1;13(13):2095. doi: 10.3390/foods13132095 (PMC11240903; doi:10.3390/foods13132095)
Supplement: Supplementary file 1 [file foods-13-02095-s001.zip › foods-3056059-supplementary.pdf]

Supplemental material

Table S1. Equations for measuring the exponential growth and doubling time for TSB, UPB, no. 17 broth and SEL for the co-culture assays.

Experiment[a]	Bacteria (CFU/mL)	TSB	UPB	no. 17	SEL
I	***E. coli* O157:H7 (1)**	Y = 0.6452X + 3.110	Y = 0.7312X – 0.2247	Y = 0.7295X – 0.3745	Y = 0.3417X + 0.07373
	***Salmonella* spp. (1)**	Y = 0.6301X + 1.219	Y = 0.6654X – 0.08246	Y = 0.5673X – 0.2385	Y = 0.2795X + 0.2667
	***L. monocytogenes* (1)**	Y = 0.3465X + 0.1060	Y = 0.3633X + 0.1605	Y = 0.3651X + 0.2481	Y = 0.2243X – 0.02190
II	***E. coli* O157:H7 (1)**	Y = 0.7058X + 0.02617	Y = 0.6881X + 0.1313	Y = 0.7242X + 0.09590	Y = 0.4425X – 0.2547
	***Salmonella* spp. (1000)**	Y = 0.6255X + 3.133	Y = 0.5979X + 3.083	Y = 0.5487X + 2.562	Y = 0.3111X + 2.719
	***L. monocytogenes* (10)**	Y = 0.3557X + 1.135	Y = 0.3199X + 1.091	Y = 0.2024X + 1.116	Y = 0.1939X + 0.7567
III	***E. coli* O157:H7 (10)**	Y = 0.7711X + 0.7909	Y = 0.7475X + 0.8142	Y = 0.6658X + 0.9479	Y = 0.3953X + 0.6898
	***Salmonella* spp. (1)**	Y = 0.6975X – 0.05564	Y = 0.6912X – 0.1061	Y = 0.6215X + 0.007388	Y = 0.3861X – 0.3540
	***L. monocytogenes* (1000)**	Y = 0.3778X + 2.941	Y = 0.3177X + 2.942	Y = 0.3249X + 3.148	Y = 0.1211X + 2.893
IV	***E. coli* O157:H7 (1000)**	Y = 0.6452X + 3.110	Y = 0.6971X + 2.876	Y = 0.5339X + 3.257	Y = 0.2596X + 2.726
	***Salmonella* spp. (10)**	Y = 0.6301X + 1.219	Y = 0.6661X + 1.088	Y = 0.6005X + 0.8814	Y = 0.3680X + 0.6707
	***L. monocytogenes* (1)**	Y = 0.3465X + 0.1060	Y = 0.3502X + 0.04121	Y = 0.2853X + 0.1802	Y = 0.2258X – 0.1814

[a] Note: *E. coli*: *Salmonella* spp.: *L. monocytogenes* ratios were as follows: experiment I - 1:1:1, experiment II - 1:1000:10, experiment III 10:1:1000, and experiment IV - 1000:10:1.

Table S2. Values of DT and μ for TSB, UPB, no. 17 broth and SEL for the co-culture assays. Table key: μ – Exponential growth rate (unit: log_{10} CFU $mL^{-1}h^{-1}$); DT – Doubling Time (unit: h); SD – Standard deviation.

		**TSB**		**UPB**		**no. 17**		**SEL**	
**Experiment[a]**	**Bacteria (CFU/mL)**	μ ± SD	DT ± SD	μ ± SD	DT ± SD	μ ± SD	DT ± SD	μ ± SD	DT ± SD
**I**	***E. coli* O157:H7 (1)**	0.65 ± 0.02	1.55 ± 0.02	0.73 ± 0.03	1.37 ± 0.01	0.75 ± 0.08	1.37 ± 0.01	0.34 ± 0.02	2.93 ± 0.10
	***Salmonella* spp. (1)**	0.63 ± 0.02	1.59 ± 0.03	0.66 ± 0.03	1.50 ± 0.02	0.58 ± 0.05	1.76 ± 0.10	0.28 ± 0.01	3.58 ± 0.15
	***L. monocytogenes* (1)**	0.35 ± 0.03	2.89 ± 0.06	0.36 ± 0.02	2.75 ± 0.09	0.37 ± 0.02	2.74 ± 0.07	0.22 ± 0.02	4.46 ± 0.07
**II**	***E. coli* O157:H7 (1)**	0.71 ± 0.03	1.42 ± 0.01	0.69 ± 0.06	1.45 ± 0.01	0.72 ± 0.02	1.38 ± 0.01	0.44 ± 0.02	2.26 ± 0.01
	***Salmonella* spp. (1000)**	0.63 ± 0.02	1.59 ± 0.03	0.59 ± 0.05	1.67 ± 0.02	0.55 ± 0.08	1.82 ± 0.04	0.31 ± 0.02	3.21 ± 0.33
	***L. monocytogenes* (10)**	0.36 ± 0.03	2.81 ± 0.12	0.32 ± 0.03	3.12 ± 0.10	0.20 ± 0.02	4.94 ± 0.40	0.19 ± 0.05	5.16 ± 0.46
**III**	***E. coli* O157:H7 (10)**	0.77 ± 0.04	1.29 ± 0.00	0.75 ± 0.04	1.34 ± 0.01	0.67 ± 0.05	1.50 ± 0.01	0.39 ± 0.04	2.53 ± 0.58
	***Salmonella* spp. (1)**	0.69 ± 0.03	1.43 ± 0.03	0.69 ± 0.04	1.45 ± 0.01	0.62 ± 0.01	1.61 ± 0.07	0.39 ± 0.04	2.59 ± 0.21
	***L. monocytogenes* (1000)**	0.38 ± 0.03	2.65 ± 0.08	0.32 ± 0.03	3.15 ± 0.11	0.32 ± 0.04	3.08 ± 0.29	0.12 ± 0.02	8.26 ± 0.37
**IV**	***E. coli* O157:H7 (1000)**	0.65 ± 0.06	1.55 ± 0.01	0.69 ± 0.05	1.43 ± 0.01	0.53 ± 0.06	1.87 ± 0.24	0.26 ± 0.06	3.85 ± 0.64
	***Salmonella* spp. (10)**	0.63 ± 0.04	1.59 ± 0.02	0.67 ± 0.03	0.59 ± 0.01	0.60 ± 0.04	1.67 ± 0.02	0.37 ± 0.03	2.72 ± 0.06
	***L. monocytogenes* (1)**	0.35 ± 0.04	2.89 ± 0.09	0.35 ± 0.02	0.24 ± 0.09	0.29 ± 0.04	3.50 ± 0.05	0.23 ± 0.04	4.43 ± 0.64

[a] Note: *E. coli*: *Salmonella* spp.: *L. monocytogenes* ratios were as follows: experiment I - 1:1:1, experiment II - 1:1000:10, experiment III 10:1:1000, and experiment IV - 1000:10:1.

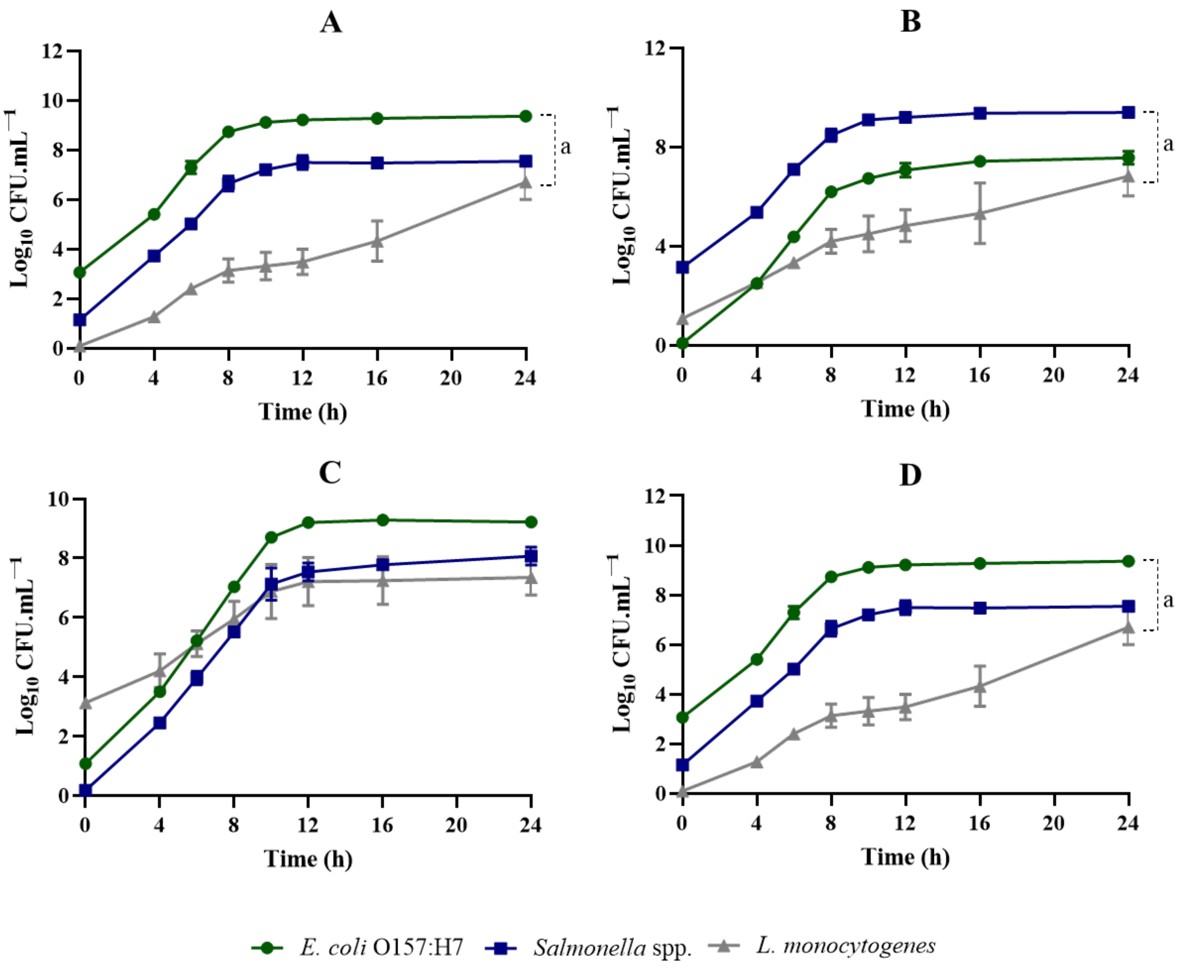

Figure S1. Growth curves for *E. coli* O157:H7, *Salmonella* spp. and *L. monocytogenes* co-culture in TSB, at different pathogen ratios. *E. coli* O157:H7/*Salmonella* spp./*L. monocytogenes* ratio of 1:1:1 **(A)**, 1:1000:10 **(B)**, 10:1:1000 **(C)** and 1000:10:1 **(D)**. Error bars represent the standard deviation of three biological replicates. [a] Indicates that there is a statistical difference in values of μ ($P < 0.05$) between *E. coli* O157:H7 and *L. monocytogenes*.

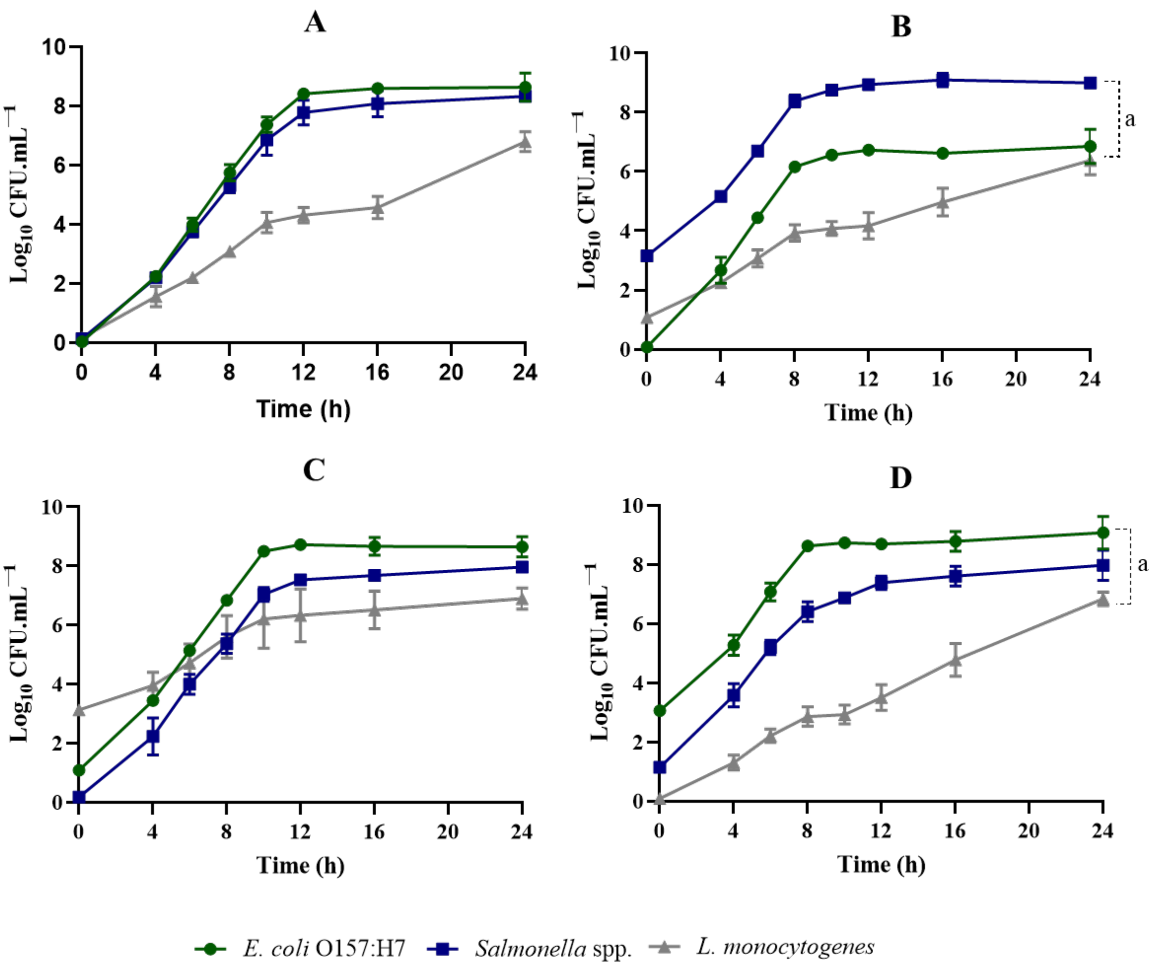

Figure S2. Growth curves for *E. coli* O157:H7, *Salmonella* spp. and *L. monocytogenes* co-culture in UPB, at different pathogen ratios. *E. coli* O157:H7/*Salmonella* spp./*L. monocytogenes* ratio of 1:1:1 **(A),** 1:1000:10 **(B)**, 10:1:1000 **(C)** and 1000:10:1 **(D)**. Error bars represent the standard deviation of three biological replicates. [a] Indicates that there is a statistical difference in values of μ ($P < 0.05$) between *E. coli* O157:H7 and *L. monocytogenes*.

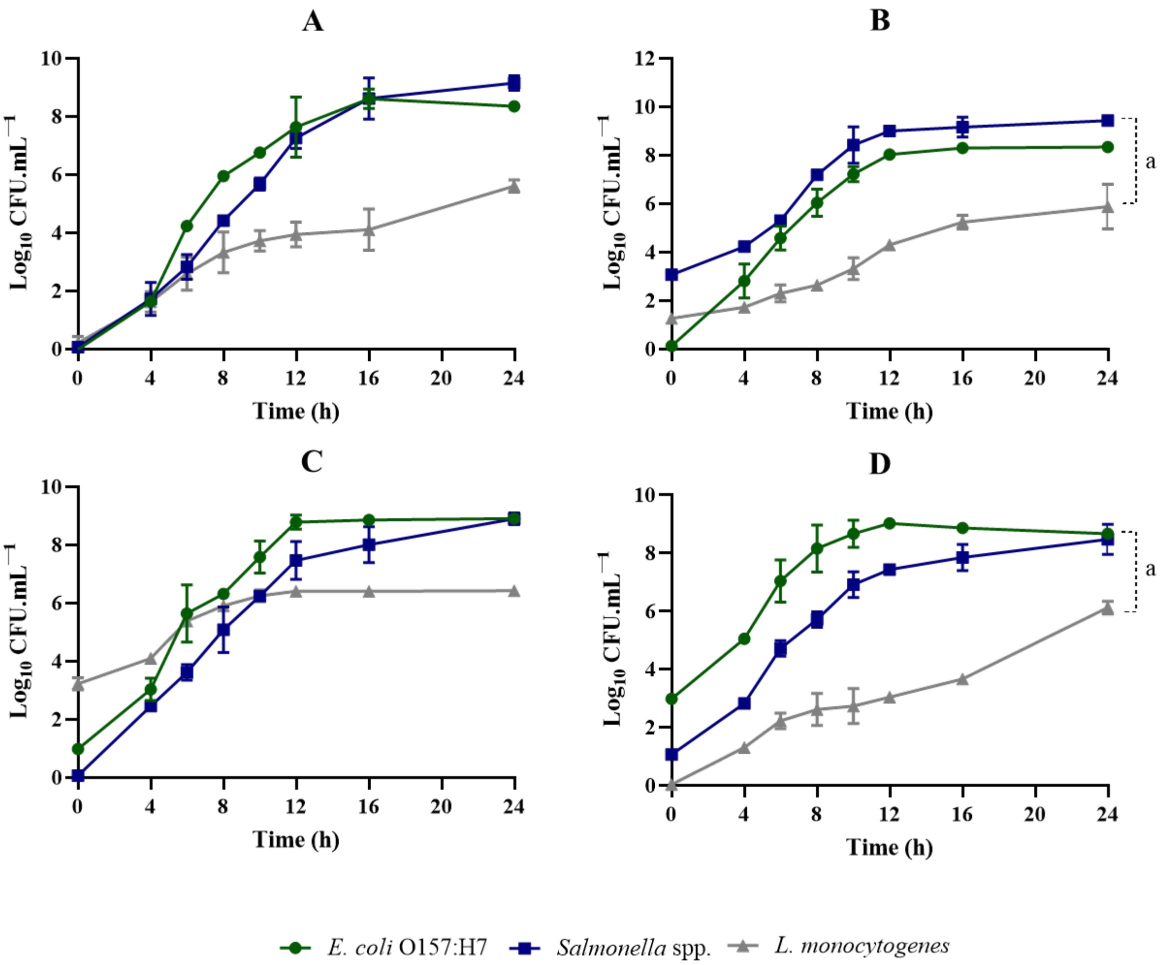

Figure S3. Growth curves for *E. coli* O157:H7, *Salmonella* spp. and *L. monocytogenes* co-culture in no.17 broth, at different pathogen ratios. *E. coli* O157:H7/*Salmonella* spp./*L. monocytogenes* ratio of 1:1:1 **(A)**, 1:1000:10 **(B)**, 10:1:1000 **(C)** and 1000:10:1 **(D)**. Error bars represent the standard deviation of three biological replicates. [a] Indicates that there is a statistical difference in values of μ ($P < 0.05$) between *E. coli* O157:H7 and *L. monocytogenes*.
